# Patient empowerment in hand hygiene

**DOI:** 10.1186/2047-2994-4-S1-P291

**Published:** 2015-06-16

**Authors:** YC Yau

**Affiliations:** 1 The Duchess of Kent Children’s Hospital at Sandy Bay, Hong Kong

## Introduction

Patient empowerment is an integral part of the WHO hand hygiene multimodal strategy. In spite of the usage of educational tools and reminder, the participation rate of patients in hand hygiene programs remains very low in wards.

## Objectives

To investigate the reasons of low participating rate in hand hygiene practice in Chinese patient group during their hospital stay.

## Methods

A survey was conducted from April 2013 to March 2014. A total of 952 orthopedic patients/family members, age 12 to 79 years old were interviewed. They have to answer the four simple questions and the reason for their answers.

1. Do you think hand hygiene is an important practice within the hospital? Why?

2. Would you request the attending doctors or nurses to clean their hands before approaching you? Why?

3. Is it difficult to ask doctors or nurses to perform hand hygiene? Why?

4. Do you find the hand hygiene poster and educational pamphlet useful? Why? These collected data will then be analyzed by our infection control nurses.

## Results

94% of the interviewee did not think hand hygiene was an important practice in the general ward. 90% of the interviewee expressed they would not request health professionals to practice hand hygiene. 99% of the interviewee said it is difficult to ask professionals to practice hand hygiene.80% of the interviewee thought that the hand hygiene promotional material was not useful to them.

Qualitative analysis identified the following main points:

It did not make much sense to them that improper hand hygiene could lead to life threatening events. They do not want to challenge the health professional. Most Chinese patients, especially over 50 would not take proactive action to ask doctors to do hand hygiene. Some expressed that the atmosphere in ward did not favor them to ask health professionals to practice hand hygiene. They think the contents of the posters and information pamphlets were for health professional instead for patients.

## Conclusion

Although patient’s engagement in hand hygiene is appealing, a few points need to be addressed when planning patient empowerment programs. Furthermore, the patient educational material should be simple and focus on patients more so that the message can be delivered to patients easily. Therefore, higher engagements rate in hand hygiene practice can be achieved.

## Disclosure of interest

None declared.

